# Whole-Genome Sequence of *Pseudomonas graminis* Strain UASWS1507, a Potential Biological Control Agent and Biofertilizer Isolated in Switzerland

**DOI:** 10.1128/genomeA.01096-16

**Published:** 2016-10-06

**Authors:** Julien Crovadore, Gautier Calmin, Romain Chablais, Bastien Cochard, Torsten Schulz, François Lefort

**Affiliations:** a Plants and Pathogens Group, Research Institute Land Nature and Environment, hepia, HES-SO University of Applied Sciences and Arts Western Switzerland, Jussy, Geneva, Switzerland; b Faculty of Engineering and Architecture, HES-SO University of Applied Sciences and Arts Western Switzerland, Delémont, Switzerland; c Philotimo SA, Geneva, Switzerland

## Abstract

We report here the whole-genome shotgun sequence of the strain UASWS1507 of the species *Pseudomonas graminis*, isolated in Switzerland from an apple tree. This is the first genome registered for this species, which is considered as a potential and valuable resource of biological control agents and biofertilizers for agriculture.

## GENOME ANNOUNCEMENT

*Pseudomonas graminis* is a nonfluorescent Gram-negative bacterium, described in 1999 (1). Initially isolated from the phyllosphere of grasses (2), it has also been found on fruits (3, 4), sugarcane (5), and apple (6). Some strains have been shown to reduce the growth of foodborne pathogens in fresh-cut apples (3, 4), melon (7), and fresh-cut lettuce (8), and to increase plant growth in sugarcane (5). A strain has proved to protect apple trees from the fire blight agent *Erwinia amylovora* (6, 9) more efficiently than the commercial strains A506 of *Pseudomonas fluorescens* and C9-1 of *Pantoea vagans. Pseudomonas graminis* bacteria have a GC content of 60 to 61% (1), are aerobic, non-spore-forming, rod-shaped (0.5 to 1.0 × 3.5 to 5.0 µm), and motile between 15 and 20°C. They grow in a temperature range of 4 to 40°C (optimum 25°C) and form yellow colonies. The strain UASWS1507 was isolated in Switzerland from a core sample made in the trunk of a *Malus pumila* tree. Initially identified as *Pseudomonas graminis* by 16S rDNA sequencing, it shared 99.9% identity with more than 20 *P. graminis* strains in the GenBank database (10). 

Genomic DNA was extracted from a pure axenic culture following an adapted protocol (11). Libraries were prepared using the TruSeq DNA PCR-Free library preparation kits (Illumina, USA). Whole-genome shotgun sequencing was carried out within one Illumina MiniSeq run at 2 × 150-bp paired-end read lengths, using a MiniSeq Mid Output kit, which provided a genome coverage of 128×. Quality control of the reads was assessed with FastQC (12). Genome assembly was computed with SPAdes Genome assembler 3.8.1 (13) yielding 71 contigs (≥200 bp.), which were arranged with BioEdit (14) and analyzed with QUAST (15). The genome total length was 5,957,886 bp, with a GC content of 60.25% and an *N*_50_ value of 344,532 bp. Automated gene annotation was carried out by the NCBI Prokaryotic Genome Automatic Annotation Pipeline (PGAAP) (16) and RAST version 2.0 (17, 18). PlasmidFinder (19) and plasmidSPAdes (20) did not detect any plasmid. The annotation with RAST identified 5,149 coding sequence genes (CDSs) distributed in 529 subsystems, whereas PGAAP identified 5,247 genes for 5,177 CDSs and 5,045 coding genes, 132 pseudogenes, and 70 RNA genes. No complete transposon or phages were found. 

As annotations confirmed the absence of toxins, superantigens, and virulence and disease genes, this bacterium could be considered for agricultural and environmental uses. It is equipped with 139 genes for chemotaxis and flagellar motility, which assures dissemination in soils. The presence of four genes involved in plant auxin synthesis is promising of plant growth–promoting rhizobacteria (PGPR) properties (21). The bacterium features resistance genes against metals (arsenic, cadmium, cobalt, copper, and zinc) and a few antibiotics (penicillin, fluoroquinolones, and streptothricin). The bacterium also owns 11 genes for nitrate and nitrite ammonification and 23 genes for ammonia assimilation but none for denitrification. Gene equipment in phosphorus metabolism (58 genes) and organic acids (43 genes) should support organic phosphate mineralization and phosphate solubilization, which are desired characteristics of PGPR (21). Additionally, 80 genes active in various degradation pathways of aromatic compounds offer growth capacities in contaminated soils.

### Accession number(s).

This whole-genome shotgun project was deposited at DDBJ/EMBL/GenBank under the accession number MDEN00000000. The version described in this paper is the first version, MDEN00000000.1. The 71 contigs have been deposited under the accession numbers MDEN01000001 to MDEN01000071.
